# Alteration of the gut microbiota and metabolite phenylacetylglutamine in patients with severe chronic heart failure

**DOI:** 10.3389/fcvm.2022.1076806

**Published:** 2023-01-10

**Authors:** Zhendong Zhang, Bin Cai, Yanzhuan Sun, Haiyan Deng, Hongwei Wang, Zengyong Qiao

**Affiliations:** ^1^Department of Cardiology, Anhui University of Science and Technology Affiliated Fengxian Hospital, Shanghai, China; ^2^Department of Cardiology, Shanghai Fengxian District Central Hospital, Shanghai, China

**Keywords:** chronic heart failure, intestinal flora, metabolites, phenylacetylglutamine, 16S rRNA sequencing

## Abstract

Chronic Heart Failure (CHF) is the end result of nearly all cardiovascular disease and is the leading cause of deaths worldwide. Studies have demonstrated that intestinal flora has a close relationship with the development of Cardiovascular Disease (CVD) and plays a vital role in the disease evolution process. Phenylacetylglutamine (PAGln) a metabolite of the intestinal flora, is one of the common chronic kidney disease toxins. Its concentrations in plasma were higher in patients with major adverse cardiovascular events (MACE) however, its variation in patients with various degrees of CHF has rarely been reported. Therefore, we collected stool and plasma samples from 22 healthy controls, 29 patients with NYHA Class III and 29 patients with NYHA Class IV CHF (NYHA stands for New York Heart Association) from the Department of Cardiology of Shanghai Fengxian District Central Hospital. Next, we analyzed these samples by performing bacterial 16S ribosomal RNA gene sequencing and liquid chromatography tandem mass spectrometry. The result shows: The Chao 1 index was significantly lower in both NYHA class III and NYHA class IV than it was in the control group. The beta diversity was substantially dissimilar across the three groups. The linear discriminant analysis effect size analysis (LEfSe) showed that the bacterial species with the largest differences were *Lachnospiraceae* in control group, *Enterobacteriaceae* in NYHA class III, and *Escherichia* in NYHA class IV. The concentration of PAGln was significantly different between CHF and control groups and increased with the severity of heart failure. Finally, the correlation analysis represented that *Parabacteroides* and *Bacteroides* were negatively correlated to brain natriuretic peptide (BNP) and PAGln; *Romboutsia* and *Blautia* adversely associated with PAGln; *Klebsiella* was positively interrelated with BNP; *Escherichia*-*Shigella* was positively correlated with PAGln and BNP; *Alistipes* was contrasted with BNP; and *Parabacteroides* was negatively correlated with the left ventricular end-diastolic diameter (LVEDD). This study presented that the intestinal flora and its metabolite PAGln were altered with different grades of CHF and illustrated the effects of the gut flora and its metabolite on CHF.

## 1. Introduction

Chronic Heart Failure (CHF) is a major health problem worldwide and is the end result of almost the entire forms of cardiovascular disease (1–3). The pathological mechanisms of CHF include hemodynamic disturbances, dysregulation of neurosecretory activation, and the relationship between the inflammatory response and the effects of the overexpression of oxidative stress (4–6). Despite recent developments in modern combination therapy strategies, the high readmission rate and poor prognosis of CHF are not promising (2, 7). Therefore, researchers have started to explore new pathogenesis and treatment pathways for heart failure, focusing on the intestinal flora to explore new methods to prevent and treat CHF. The intestinal flora is composed of trillions with bacteria within the gastrointestinal tract. Its role in maintaining good health, absorbing nutrients, creating hormones and vitamins, building the mucosal barrier, regulating the immune system and inhibiting the colonization of pathogenic bacteria is crucial (8–11). Studies have shown that reduced cardiac output and systemic organ system congestion can lead to edema and localized ischemia in the intestine, resulting in raised intestinal bacterial translocation and circulating endotoxin levels. This induces inflammation-related cytokine production, which in turn can promote inflammatory responses and trigger cardiac fibrosis and microvascular and cardiac dysfunctions, thereby aggravating heart failure (12–15). For the past few years, several researches have shown that the component and function of the gut flora are different in heart failure patients and healthy persons. Phenylacetylglutamine (PAGln), one of the common chronic kidney disease toxins, was previously thought to only be produced by the combination of phenylacetate/phenylbutyrate (phenylacetate precursor) and glutamine, which acts as an ammonia-lowering agent via the urea cycle bypass pathway (16). In contrast, recent studies have found that PAGln, which can also be generated through the metabolism of the essential amino acid phenylalanine by intestinal microorganisms, has high plasma concentrations in patients with Major Adverse Cardiovascular Events (MACE) and is considered to be an independent predictor of MACE risk (17, 18). However, there are few studies on the changes in the PAGln concentration in the plasma of CHF patients. Thus, in this research, we selected patients of different grades in CHF and healthy controls as the study subjects. By sequencing their fecal flora genes and detecting the PAGln levels in their plasma, we investigated (1) the relationship between intestinal flora diversity in patients with varying grades in CHF and that in the healthy controls, (2) the changes in the PAGln levels in patients with varying grades in CHF and the healthy controls, and (3) the effects of intestinal flora diversity and PAGln on CHF.

## 2. Materials and methods

### 2.1. Study subject and sample selection

We selected 58 patients of CHF hospitalized in the department of cardiology of Shanghai Fengxian District Central Hospital from June 2021 to June 2022 for this study (CHF group). There were 26 male cases and 32 female cases, aged 52–89 years. In terms of comorbid diseases, there were 22 patients with coronary heart disease, 3 patients with hypertension, and 33 patients with coronary heart disease combined with hypertension. According to the New York Heart Association (NYHA) cardiac function classification, 29 patients were categorized as class III and 29 patients were categorized as class IV. Furthermore, 22 patients with the same CHF risk factors but without clinical manifestations and a previous medical history of CHF were chosen as the control group. There were 13 male patients and 9 female patients, aged 65–86 years. In terms of comorbid diseases, there were 9 patients of coronary heart disease and 13 patients of coronary heart disease combined with hypertension. The research was authorized by the Ethics Board of Shanghai Fengxian District Central Hospital, and all the patients (or their direct relatives) signed the informed consent form.

The exclusion standards included (1) hypertrophic or restrictive cardiomyopathy, myocarditis, acute myocardial infarction, and congenital heart disease; (2) diabetes mellitus, diarrhea, Crohn’s disease, ulcerative colitis, infectious diseases, and malignancy; (3) severe hepatic and renal insufficiency; (4) having taken antibiotics, steroid hormones, herbal medicines (including oral, intramuscular, or intravenous), or probiotics (such as yogurt) in the 3–6 months before admission; and (5) a substantial change in diet in the week before admission.

### 2.2. Phenylacetylglutamine detection method

We collected 5 ml of venous blood within 24 h of admission from both groups, the samples were mixed in EDTA anticoagulation tubes, and 1 ml of plasma was separated and preserved at −80°C in an ultra-low temperature refrigerator. The plasma PAGln level was measured through liquid chromatography tandem mass spectrometry. The plasma samples (100 μL) were vortexed with methanol (400 μL), sonicated and centrifuged (12,000 rpm, 4°C, 10 min). The supernatant was subsequently removed and analyzed on the machine. Next, chromatographic separation was performed on a column Acquity UPLC HSS T3 1.8 μm (Waters, Milford, USA) with the following conditions: flow rate, 0.30 ml/min; column temperature, 40°C; injection volume, 5 μl; mobile phase composition, A, water (0.1% formic acid) and B, methanol; and sample gradient elution program, 0–1.0 min 10% B. The PAGln was detected using an AB Sciex Triple Quad 5,500 mass spectrometer(AB SCIEX, Framingham, USA) with the setting thermoelectric spray ion source mode. The data were collected using the multiple reaction monitoring mode with the parameters 263.2 Da for Q1 Mass and 145.2 Da for Q3 Mass. The declustering potential was −29 V, and the collision energy was −15 V. The MultiQuant software was used for the integration, and the PAGln content was calculated using the standard curve.

### 2.3. Biological analysis

#### 2.3.1. DNA extraction

We collected 5.0 g of fresh early morning stools from patients in the CHF and control groups within 24 h after admission in sterile tubes, and the sampling tubes were placed in self-sealing bags. The samples were delivered to the laboratory within 2 hours and stored in an ultra-low temperature refrigerator set at −80°C. The DNA extraction for the microbial community was performed according to the instructions of the DNA Extraction Kit (DP712), and the purity and concentration of the DNA were tested using a Thermo NanoDrop One after DNA extraction.

#### 2.3.2. Polymerase chain reaction amplification and product purification

The regions V3 to V4 of the bacterial 16S ribosomal RNA (rRNA) gene were amplified by the primers 338F (5′-ACTCCTACGGGAGGCAGCAG-3′) and 806R (5′-GGACTACHVGGGTWTCTAAT-3′). The polymerase chain reactions (PCRs) were performed in a volume of 50:25 μl of 2 × Premix Taq, 1 μL of the forward and reverse primers, and 50 ng of DNA. The PCR cycling was performed at a BioRad S1000 thermal cycler (Bio-Rad Laboratories, CA, USA). The reaction conditions of the PCR system were 94°C pre-denaturation for 5 min; 30 cycles of denaturation at 94°C for 30 s, annealing at 52°C for 30 s, extension at 72°C for 30 s; and finally extension at 72°C for 10 min. The PCR products were purified by electrophoresis on 1% agarose gels. Product concentrations of PCR were compared using GeneTools analysis software (version 4.03.05.0, SynGene). The volume needed for each sample was computed based on the equal mass principle and each PCR product was mixed. The PCR mix was recovered by using the EZNA Gel Extraction Kit (Omega, USA), and the target DNA fragments were recovered with elution with TE buffer.

#### 2.3.3. Library preparation and sequencing

The library were constructed following the standard procedure of the NEBNext Ultra II DNA Library Prep Kit for Illumina (New England Biolabs, Ipswich, USA). The raw sequences were then sequenced using an Illumina Nova 6000 platform for PE250 sequencing, and the sequencing data were processed using fastp (0.14.1) software. The sequence information for the different treatment groups was clustered into Operational Taxonomic Units (OTUs) for species classification according to the UPARSE clustering method, and the similarity of the OTUs was set to 97%. The species annotation information was obtained by comparing them with the SILVA (16S) database.

### 2.4. Study approval

Informed and written agreement was acquired from all patients before the trial. The Ethics Committee of Shanghai Fengxian District Central Hospital approved the experimental protocol. The trial was registered at trialos.com (SL2021-KY-25-02).

### 2.5. Statistical analysis

Enumeration data were compared with *Chi*-square test. For the normally distributed measures, the data from two groups were compared using the independent samples t-test; and the data from multiple groups were compared by the one-way ANOVA test. For the non-normally distributed measures, the data from two groups were compared by using a Mann-Whitney U test, and the data from multiple groups were compared by using the Kruskal-Wallis test. Spearman’s rank correlation was employed to analyze the correlation between variables. All the statistical analyses were performed on SPSS (version 26.0), and the significance was setting to *p* < 0.05. The alpha-diversity (Shannon and Chao1), non-metric multidimensional scaling, redundancy analysis/canonical correlation analysis (CCA), and correlation analysis were determined on the Magigene Cloud Platform^1^.

## 3. Results

### 3.1. Characteristics of the study participants

The general baseline characteristics of all participants are presented in Table 1. There were no statistically substantial distinctions in the age, composite risk factors (coronary heart disease and hypertension), smoking status, systolic blood pressure, white blood cell, high-density lipoprotein and low-density lipoprotein levels, and medication use (angiotensin-converting enzyme inhibitor/angiotensin receptor antagonist and β-blockers) between the three groups (*p* > 0.05). Heart rate, serum creatinine, blood urea nitrogen, and diuretics were significant differences across the three groups (*p* < 0.01).

**TABLE 1:** Baseline characteristics of the study participants.

Variables	Control (*n* = 22)	NYHAIII (*n* = 29)	NYHAIV (*n* = 29)	*p*
Age, years	76.0 (73.75–80.0)	77.0 (73.5–83.5)	79.0 (69.5–86.5)	0.594
Cardiovascular disease	22 (100%)	28 (96.5%)	27 (93.1%)	0.775
Hypertension	13 (59.0%)	19 (65.5%)	17 (58.6%)	0.839
Atrial fibrillation	0(0.0%)	18 (62.0%)	17 (58.6%)	<0.001
Smoking	4 (18.1%)	5 (17.2%)	9 (31.0%)	0.385
SBP/mmHg	137.5 (125.25–143.25)	132 (122.5–143.5)	128 (117.5–141.0)	0.504
Heart rate/bpm	77.5 (73.5–81.5)	78.0 (69.5–94.5)	89.0 (81.0–99.0)**^△△^	<0.01
WBC/(10^9^⋅L^–1^)	5.9 (5.29–7.19)	5.65 (4.53–8.68)	5.76 (4.71–8.00)	0.995
HDL–C/(mmol⋅L^–1^)	1.14 (1.03–1.42)	1.09 (0.93–1.36)	1.08 (0.80–1.30)	0.315
LDL–C/(mmol⋅L^–1^)	2.45 (1.99–3.01)	2.41 (1.92–3.18)	2.20 (1.60–2.60)	0.214
Triglycerides/ (mmol⋅L^–1^)	0.95 (0.70–1.38)	0.79 (0.62–1.09)	0.78 (0.64–1.14)	0.353
Serum creatinine/ (μmol⋅L^–1^)	61.5 (52.5–76.5)	81.0 (68.5–106.5)**	98.0 (78.5–148.0)**	<0.001
BUN/(mmol⋅L^–1^)	4.95 (4.08–6.10)	6.80 (5.45–7.55)*	9.70 (8.05–16.0)**^△△^	<0.001
ACEI/ARB	10 (45.4%)	13 (44.8%)	9 (31.0%)	0.466
β blocker	10 (45.4%)	18 (62.0%)	12 (41.3%)	0.255
Diuretics	3 (13.6%)	29 (100%)**	29 (100%)**	<0.001

SBP, systolic blood pressure; WBC, white blood cell; HDL-C, high density lipoprotein-cholesterol; LDL-C, low density lipoprotein-cholesterol; BUN, blood urea nitrogen; ACEI, angiotensin converting enzyme inhibitor; ARB, angiotensin receptor antagonist. When compared with the control group, *p < 0.05, **p < 0.01; when compared with NYHA class III, ^△△^*p* < 0.01.

### 3.2. Comparison of the echocardiographic indices, brain natriuretic peptide, and phenylacetylglutamine levels between the three groups

The comparison of the echocardiographic indices, brain natriuretic peptide (BNP), and PAGln levels of all the participants is shown in Table 2. Regarding the echocardiographic indices, left ventricular ejection fraction (LVEF), left ventricular end-systolic diameter (LVESD), left ventricular end-diastolic diameter (LVEDD), BNP and PAGln were statistically significantly different in the three groups(*p* < 0.001). The difference in LVEF between the three groups was statistically significant (*p* < 0.01). Pairwise comparison showed significant difference. Additionally, the differences in LVESD and LVEDD between the control group and NYHA class IV were statistically significant (*p* < 0.01), LVESD and LVEDD between NYHA class III and NYHA class IV were statistically significant (*p* < 0.01), and the differences in LVESD and LVEDD between the control group and NYHA class III were not statistically significant (*p* > 0.05). Also, the results of the comparison between each paired group showed that the differences in the BNP and PAGln levels in the three groups were statistically significant (*p* < 0.01).

**TABLE 2:** Comparison of the echocardiographic indicators, brain natriuretic peptide (BNP), and phenylacetylglutamine (PAGln) levels between Control, New York Heart Association (NYHA) class III, and NYHA class IV.

Variables	Control (*n* = 22)	NYHAIII (*n* = 29)	NYHAIV (*n* = 29)	*p*
LVEF/%	67.0 (62.0–71.3)	63.0 (42.5–66.5)*	43.0 (38.5–50.0)**^△△^	< 0.001
LVESD/mm	29.0 (26.0–31.0)	31.0 (27.5–44.5)	45.0 (36.0–51.0)**^△△^	< 0.001
LVEDD/mm BNP/(ngmL^–1^)	45.5 (43.0–48.3) 42.53 (15.5–102.7)	48.0 (43.0–57.0) 1014.7 (802.6–1321.0)**	59.0 (50.5–63.0)**^△△^ 2789.2 (2256.8–3781.6)**^△△^	< 0.001 < 0.001
PAGln/(ngmL^–1^)	28.0 (12.9–44.3)	104.0 (77.2–115.7)**	186.7 (142–1340.8)**^△△^	< 0.001

LVEF, left ventricular ejection fraction; LVEDD, left ventricular end diastolic diameter; LVESD, left ventricular end systolic diameter. When compared with the control group, *p < 0.05, **p < 0.01; when compared with NYHA class III, ^△△^*p* < 0.01.

### 3.3. Spearman correlation analysis

In Table 3,the Spearman correlation analysis showed that BNP (*r* = 0.924, *p* < 0.001), LVESD (*r* = 0.529, *p* < 0.001), and LVEDD (*r* = 0.477, *p* < 0.001) were positively correlated with PAGln, and LVEF (*r* = −0.587, *p* < 0.001) was negatively correlated with PAGln.

**TABLE 3:** Spearman correlation analysis of phenylacetylglutamine and the clinical data.

Variables	*r*	*p*
BNP	0.924	<0.001
LVEF	-0.587	<0.001
LVESD	0.529	<0.001
LVEDD	0.477	<0.001

BNP, brain natriuretic peptide; LVEF, left ventricular ejection fraction; LVEDD, left ventricular end diastolic diameter; LVESD, left ventricular end systolic diameter.

### 3.4. Species community analysis

**FIGURE 1:**
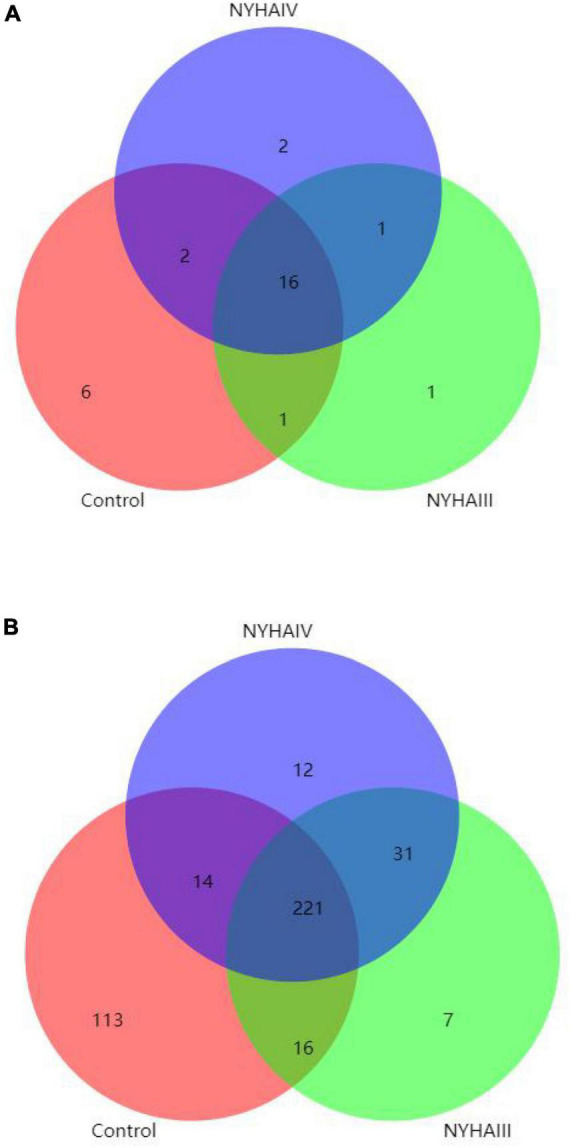
Statistical analysis of the shared and unique Operational Taxonomic Units between Control, New York Heart Association (NYHA) class III, and NYHA class IV. **(A)** Phylum level. **(B)** Genus level.

The species composition and similarity of the intestinal flora in the CHF and control groups were compared using a Venn diagram. As Figure 1 shows, at the phylum level, the total number of OTUs for the three groups was 16, the number of NYHA class III-specific OTUs was 1, the number of NYHA class IV-specific OTUs was 2, and the number of control group-specific OTUs was 6. At the genus level, the total number of OTUs in the three groups was 221, the number of NYHA class III-specific OTUs was 7, the number of NYHA class IV-specific OTUs was 12, and the number of control group-specific OTUs was 113. In addition, the number of endemic OTUs was significantly lower in NYHA class III and NYHA class IV than in the control group. The result indicates that the degree of microbial diversity in the CHF group was lower than that in the control group.

### 3.5. Diversity and strain type analysis

**FIGURE 2:**
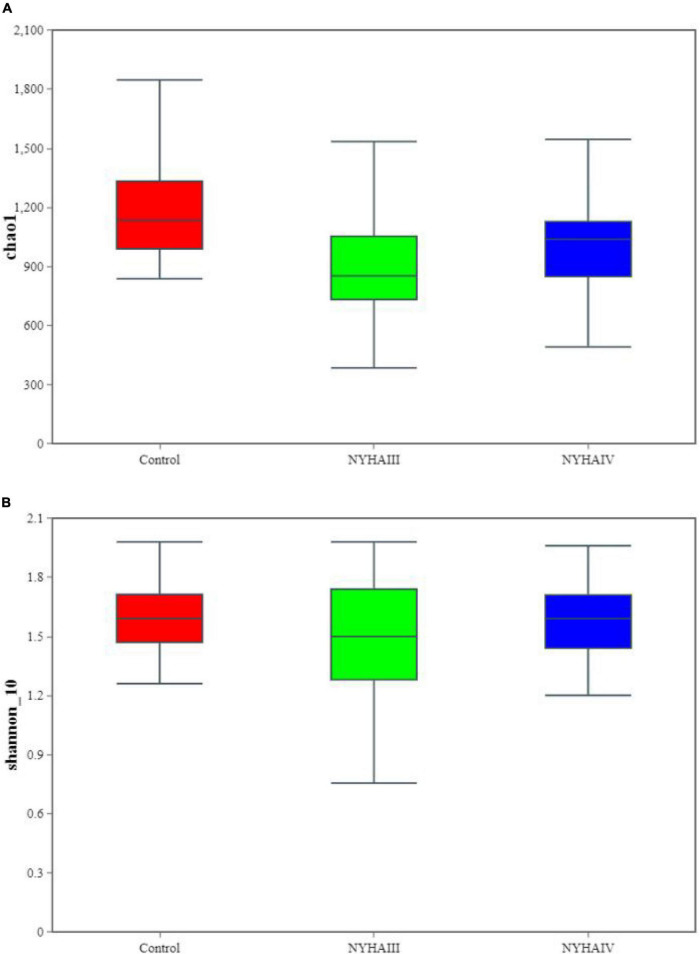
The α-diversity index analysis between Control, New York Heart Association (NYHA) class III, and NYHA class IV(at the Operational Taxonomic Unit level). **(A)** Chao1 index. **(B)** Shannon index.

**FIGURE 3:**
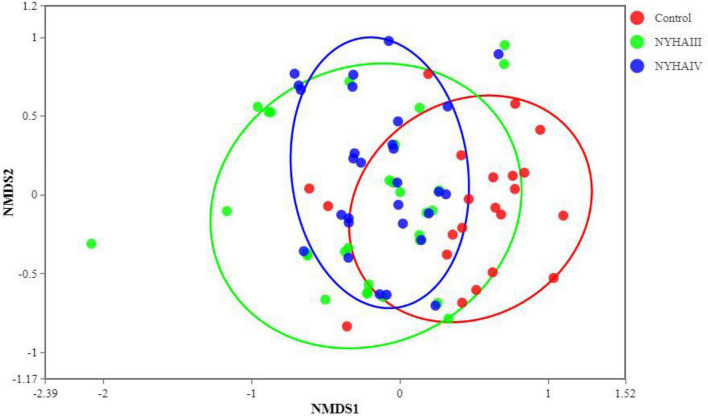
Non-metric multidimensional scaling (NMDS) analysis among Control, New York Heart Association (NYHA) class III, and NYHA class IV.

**FIGURE 4:**
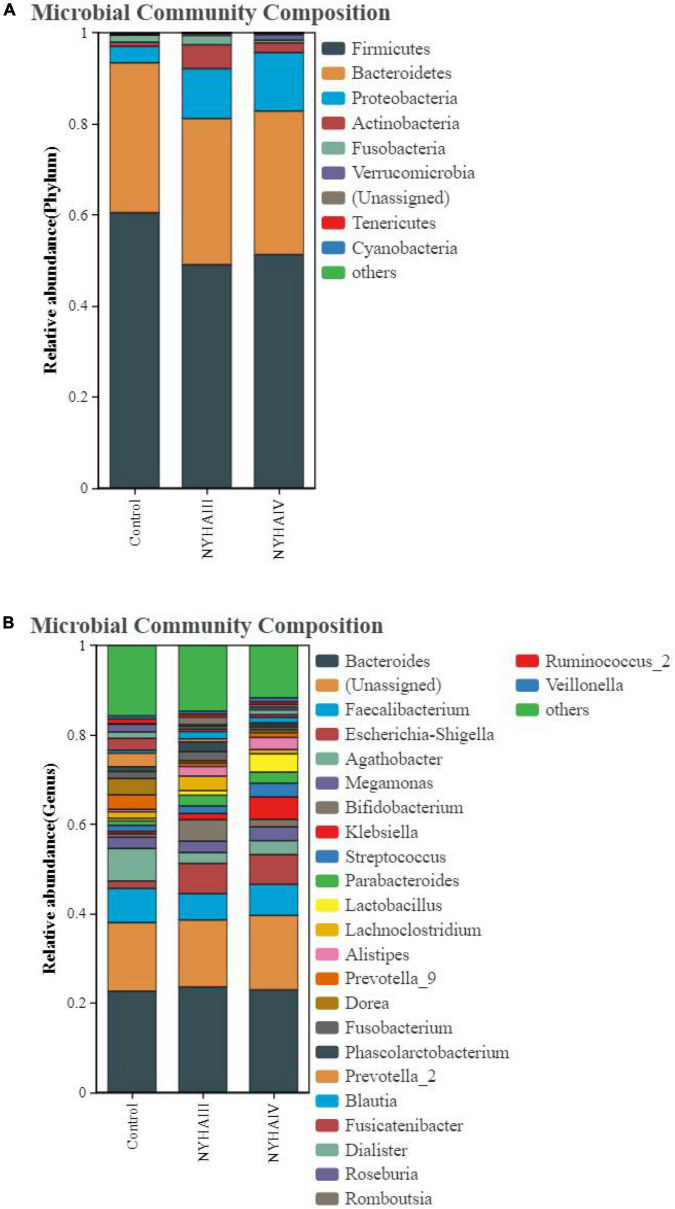
The relative abundance distribution map of Control, New York Heart Association (NYHA) class III, and NYHA class IV of bacteria. **(A)** Phylum level. **(B)** Genus level.

**FIGURE 5:**
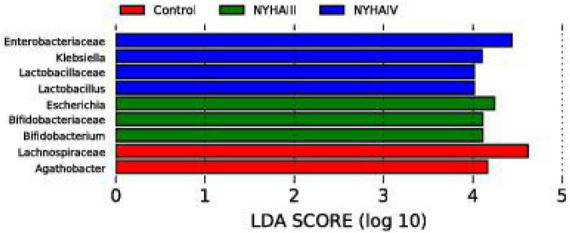
The linear discriminant analysis (LDA) effect size analysis of Control, New York Heart Association (NYHA) class III, and NYHA class IV of bacteria with an LDA score > 4.

Wilcox rank sum test was used to analyze the significance of the differences in the α-diversity index (Chao 1, Shannon) of the three groups. As shown in Figure 2, the difference in the Chao 1 index between NYHA class III and NYHA class IV was found to be not statistically significant (*p* > 0.05). The Chao 1 index was lower in both NYHA class III and NYHA class IV than in the control group, and the difference was statistically significant(*p* < 0.05). The above results indicated that the more severe the degree of heart failure, the lower the number of flora species in heart failure patients. Furthermore, the Shannon index was not statistically significant (*p* > 0.05) among the control, NYHA class III, and NYHA class IV groups, indicating that the intestinal flora diversity was essentially the same in all three groups of patients. Non-metric multidimensional scaling is a method that is used to characterize the differences in microbial communities by reducing the dimensionality of the sample distance matrix, simplifying the data structure, and showing the distribution of the samples at specific distances. It can be used to analyze the dissimilarity in the microbial community composition among the three groups of samples. As shown in Figure 3, the sample points in the heart failure and control groups were distinctly separated, and NYHA III and NYHA IV had certain degree of overlap. The sample points were clustered within the respective ranges of the three groups, indicating some discrepencies in the composition of the intestinal flora of the three groups. Community composition analysis can show the proportion of the species composition of different samples at various taxonomic levels and reflect the community structure of samples at each taxonomic level. As shown in Figure 4, at the phylum level, the most predominant taxon in the control, NYHA class III, and NYHA class IV was Firmicutes, with statistically a significant difference among the three groups (60.5:48.8:51.1%, *p* < 0.05). *Bacteroidetes* was the second most represented strain in the three groups, with 32.8, 32.8, and 32.1% in the control, NYHA class III, and NYHA class IV respectively, with no statistically significant difference among the three groups (*p* > 0.05). The percentage of the remaining phyla, such as *Proteobacteria* and *Actinobacteria*, was relatively low. The abundance percentages of *Escherichia-Shigella*, *Agathobacter*, *Megamonas*, *Bifidobacterium*, *Klebsiella*, *Streptococcus*, and *Lactobacillus* in the three groups were significantly different at the genus level (*p* < 0.05). Linear discriminant analysis effect size (LEfSe) was used to conduct the colony difference analysis to find the biomarkers. The method integrates statistical analysis of the differences and the score of the influence of that difference on the grouping results while emphasizing statistical significance and biological relevance. The results are shown in Figure 5. As shown in Figure 5, the bacterial species with the largest differences were *Lachnospiraceae* in control group, *Enterobacteriaceae* in NYHA class III, and *Escherichia* in NYHA class IV.

### 3.6. Environmental factor association analysis

**FIGURE 6:**
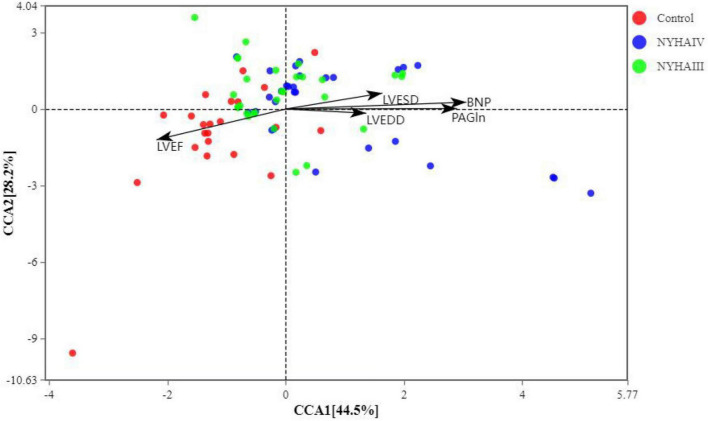
Canonical correlation analysis (CCA) of the samples (dots) and clinical data (arrows). The black arrows indicate the clinical phenotype data and their lengths indicate the degree of influence of the indicator on the gut flora.

**FIGURE 7:**
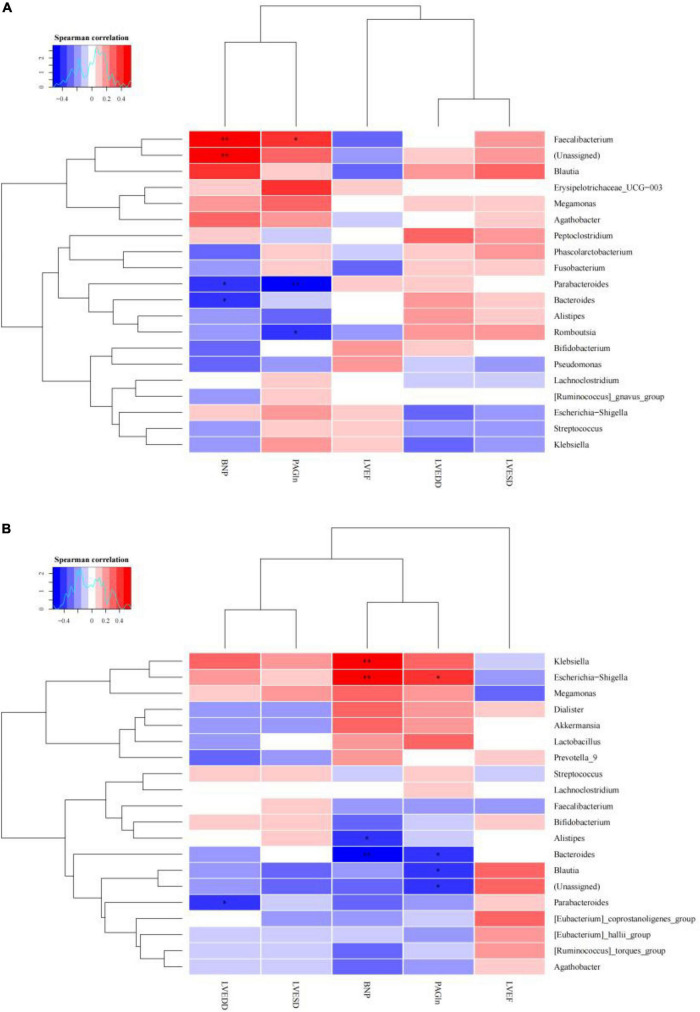
Correlation heat map of the dominate microbes and clinical data concentration (*p < 0.05, ** *p* < 0.01). **(A)** New York Heart Association (NYHA) class III. **(B)** NYHA class IV.

A CCA analysis can explain the relationship between clinical phenotypic data (such as PAGln in this study) and the gut microbial community (Figure 6). The total interpretation of the species distribution by CCA1 and CCA2 reached 72.7%. This result shows that stool samples from heart failure and controls can be better classified within the limitations of the data. It indicates that there is a significant influence of heart failure on the intestinal flora. Figure 6 shows that BNP, PAGln, LVEDD, and LVESD were significantly correlated with the CHF group of the colonies, with BNP having the greatest effect on community change. The correlation analysis (also known as Spearman correlation analysis), was performed by calculating the Spearman correlation coefficients between the clinical phenotype data and species abundance to obtain the correlation and significance between them. We selected the top 20 species in terms of abundance at the genus level for the correlation analysis with the clinical data. As shown in Figure 7, *Parabacteroides* and *Bacteroides* were negatively correlated with BNP and PAGln, and *Romboutsia* and *Blautia* were negatively correlated with PAGln. Moreover, *Klebsiella* was positively correlated with BNP, and *Escherichia-Shigella* was positively correlated with BNP and PAGln. *Alistipes* was negatively correlated with BNP, and *Parabacteroides* was negatively correlated with LVEDD.

## 4. Discussion

Tang et al. (14) put the “gut hypothesis of heart failure” forward for the first time. The hypothesis indicates that decreased cardiac production owning to heart failure can bring about reduced intestinal perfusion, mucosal ischemia, and intestinal mucosal destruction. These alterations in the intestinal barrier function, in turn, can result in raised intestinal permeability, intestinal malnutrition, bacterial translocation, and lifted circulating endotoxins, leading to potential inflammation that is connected with CHF. To minimize confounding factors, we recruited patients with age, sex, and cardiovascular disease similar to that of CHF patients as control group. In this study, we found that although there was no substantially difference in the CHF risk factors (coronary heart disease and hypertension) among the three groups, the three groups had significantly different intestinal flora structures as determined by the 16S rRNA amplification sequence assay. A comparison of the distribution of the OTUs in all three groups at the phylum level disclosed that the amount of OTUs specific to the control group was 20.7%, the number of OTUs specific to NYHA class III was 3.4%, and the number of OTUs specific to NYHA class IV was 6.7%. At the genus level, the number of control-specific OTUs was 27.2%, the number of NYHA class III-specific OTUs was 1.7%, and the number of NYHA class IV-specific OTUs was 2.9%. Therefore, the number of NYHA class III and NYHA class IV unique OTUs was considerably less than that of the control group, indicating that the microbial diversity degree in the CHF group was lower than that in the control group. By comparing the microbial diversity of the three groups, we found that the Chao 1 index was lower in both NYHA class III and NYHA class IV than that in the control group (*p* < 0.05). According to the above results, the more severe the level of heart failure, the lower the number of flora species in heart failure patients. The β-diversity analysis revealed significant separation between the three groups of sample points and clustering within a certain range, indicating differences in the gut flora composition between the three groups. This indicates that there is a connection in the severity of heart failure and the distribution and components of the gut flora. The research demonstrated that the intestinal perfusion of CHF patients was insufficient, and the intestinal structure and function were changed, causing a clear reduction in the diversity of the intestinal flora (19). Additionally, in terms of the species composition and difference analysis, we identified statistically marked differences in the Firmicutes abundance among the three groups at the phylum level (60.5:48.8:51.1%, *p* < 0.05), with a marked reduction in the Firmicutes abundance in the CHF group. It was also discovered that *Bacteroidete*’s abundance was raised and *Firmicute*’s abundance was reduced in the CHF patients, and the abundance of Firmicutes and intestinal microbial diversity were positively correlated with the intake of dietary fiber (20). However, CHF patients generally had digestive symptoms such as nausea due to long-term gastrointestinal stasis, which led to insufficient dietary fiber intake and led to a reduction of the abundance of thick-walled bacteria phyla. Thus, we consider that the decrease in the abundance of Firmicutes is one of dysbiosis’ typical manifestations in CHF patients. In terms of the species composition and analysis of variance, we found that the control, NYHA class III, and NYHA class IV *Escherichia-Shigella* (1.7:7.2:6.6), *Bifidobacterium* (0.8:4.2:1.6), *Klebsiella* (0.5:1.34:4.7), and *Lactobacillus* (0.6:1.17:4.28) had significantly different abundance shares at the genus level (*p* < 0.05). The LEfSe analysis demonstrated that at the genus level, *Agathobacter* was significantly higher in the control, and *Escherichia* and *Bifidobacterium* were significantly higher in NYHA class III. Then, *Klebsiella* and *Lactobacillus* were significantly higher in NYHA class IV. The genera that were statistically different in both test analyses were *Bifidobacterium*, *Klebsiella*, and *Lactobacillus*. Moreover, *Bifidobacterium* was higher in NYHA class III than that in NYHA class IV, and *Klebsiella* in NYHA class III was lower than that in NYHA class IV. This suggests that *Bifidobacterium* and *Klebsiella* may be the key organisms that change under different degrees of CHF. Studies have shown that *Klebsiella* is elevated in cardiovascular disease and that it regulates the trimethylamine synthesis pathway, which can be metabolized by intestinal microbes to produce trimethylamine N-oxide (TMAO). Additionally, researches have found that elevated plasma TMAO levels in CHF patients predict a higher risk of death (21). The Choline-TMA-TMAO production pathway can directly contribute to the development of adverse ventricular remodeling and heart failure. It was found that mice fed with a high choline diet had higher TMAO levels and were prone to ventricular remodeling. The degree of ventricular diastole, ventricular wall thinning, and myocardial fibrosis were significantly increased compared to the control group, and ventricular wall contractility was significantly decreased (22). Furthermore, animal models have shown that *Lactobacillus plantarum* Lp299v-containing beverages can reduce myocardial hypertrophy and ventricular remodeling, and *Lactobacillus rhamnosus* GR-1 could significantly improve the prognosis of rats with ischemic heart failure (23). Wang et al. found that *Bifidobacterium* can extensively reduced plasma TMAO levels in choline-fed mice (24). Meanwhile, *Bifidobacterium* exerts various physiological effects as a beneficial bacterium, including reducing the number of harmful bacteria (such as *E. coli*), inhibiting the generation of pro-inflammatory cell factors, regulating host organism immunity, and enhancing the gut environment by depressing ammonia concentration in feces and intestinal pH. Therefore, the increased abundance of *Bifidobacterium* with heart failure could be the consequence of a feedback mechanism of the organism, acting against pathogenic bacteria and toxic mediators. For instance, some studies have shown an increased abundance of *Bifidobacterium* in heart failure patients (25). This is consistent with our findings and indicates that alterations in the structural composition of the gut flora might be a potential factor in the progression of CHF.

Phenylacetylglutamine is a byproduct of the catabolism of the essential amino acid phenylalanine by intestinal microorganisms. Phenylalanine is catabolized by the gut flora to form phenylpyruvate and phenylacetic acid, and liver enzymes catalyze the formation of PAGln from phenylacetic acid and glutamine (26, 27). The PAGln plasma concentration was found to independently predict the risk of MACE (18). Then, BNP is the most widely used bioactive hormone for the diagnosis in heart failure. Also, LVEDD and LVESD are common clinical echocardiographic indices that can effectively reflect structural changes in the left ventricle ejection fraction. The Spearman correlation analysis in this study showed that BNP (r = 0.924, *p* < 0.001), LVESD (r = 0.529, *p* < 0.001), LVEDD (r = 0.477, *p* < 0.001) were positively correlated with PAGln, and LVEF (r = -0.0.587, *p* < 0.001) was negatively correlated with PAGln. The CCA analysis showed that five significant clinical phenotypic data explained 72.7% of the species distribution. The arrows representing BNP and PAGln had the longest lengths, indicating that BNP and PAGln had the greatest effect on the change in the flora; the BNP and PAGln arrows had similar lengths, indicating that both have similar effect sizes on the flora of the CHF group. Hyperactivation of the sympathetic nervous system is a distinctive characteristic of CHF, and prolonged activation can lead to the worsening of CHF. Thus, PAGln can act on the β2-adrenergic receptors in the heart, resulting in hyperexcitation of the sympathetic nervous system, which can exacerbate heart failure (28). PAGln also was substantially different among patients who did and did not experience heart failure-related events (29). Therefore, the level of PAGln can be considered an indicator for screening for heart failure and predicting prognosis.

In our study, the plasma PAGln concentrations were substantially higher in the CHF group compared to the control group(*p* < 0.001), and the plasma PAGln concentrations were substantially higher in the patients in NYHA class IV than those in NYHA class III (*p* < 0.001), indicating that the more severe the degree of heart failure, the higher the PAGln concentration. The correlation analysis revealed that *Parabacteroides* and *Bacteroides* were negatively correlated with BNP and PAGln, and *Romboutsia* and *Blautia* were negatively correlated with PAGln. *Klebsiella* was positively correlated with BNP, and *Escherichia-Shigella* was positively correlated with BNP and PAGln. Furthermore, *Alistipes* was negatively correlated with BNP, and *Parabacteroides* was negatively correlated with LVEDD. Inflammation and myocardial fibrosis are the basic pathological mechanisms of heart failure. With heart failure, there is a lack of intestinal motility, impaired absorption in the mucosal villi, and damage to the mucosal barrier of the intestinal epithelium, which subsequently leads to increased intestinal permeability and failure to remove bacteria and food residues from the intestinal lumen promptly. This promotes the proliferation of Gram-negative bacteria such as *Klebsiella*, resulting in the easy entry of toxic mediators such as lipopolysaccharides (LPS) into the circulation, further stimulating the activation of the inflammation and immune response and exacerbating the progression of heart failure. Therefore, we suggest that *Klebsiella* exacerbates the progression of heart failure by releasing LPS. Also, we speculate that PAGln is a microbial metabolite that is present in *Klebsiella*, and the role and mechanism of *Klebsiella* in the development of heart failure should be investigated more thoroughly in the future. In addition, there were significant differences in the LVEF, LVESD, LVEDD, and BNP indices across the three groups. These indices are often involved in the diagnosis and differential diagnosis of heart failure, assessing the extent of the disease, and evaluating the prognosis, and are widely used in clinical practice. In mice given an ob/ob and high-fat diet, Wang et al. (30) reported that *Parabacteroides distasonis* had positive metabolic effect by decreasing weight gain, insulin resistance, and hepatic steatosis. Interventional treatment of mice fed a high-fat diet using *P. distasonis* revealed that *P. distasonis* dramatically altered the bile acid profile, elevating the content of lithocholic acid and ursodeoxycholic acid and increasing the level of succinic acid in the intestine. Additionally, *Romboutsia* species, such as *Romboutsia sedimentorum* (31) and *Romboutsia ilealis* (32), were capable to apply carbohydrates and glucose to create short-chain fatty acids(SCFAs). In a mouse model, SCFAs inhibited hypertension-induced cardiac inflammatory responses, myocardial hypertrophy, and fibrosis (33–35). Previous studies have found that corticosterone can combine with and activate mineralocorticoid receptors, leading to sodium retention, which can lead to increased blood pressure (36, 37). A study (38)showed that the intestinal corticosterone levels were increased 4.4-fold in Wistar rats on a high salt diet. This study also found that corticosterone is significantly and negatively associated with some high abundance bacteria such as *Bacteroides* and *Alistipes*. *Bacteroides* can create arachidonic acid to prevent intestinal corticosterone synthesis and cut down intestinal corticosterone’s elevation owning to a high-salt diet, therefore, exerting a hypotensive effect. Previous studies have found a reduced abundance of *Blautia* in patients with CHF (19). *Blautia* is one of the important SCFA-producing bacteria, with anti-inflammatory effects, which contributes to intestinal mucosal injury’s recovery and is correlated with the visceral fat content inversely (39). Then, Empagliflozin improved cardiovascular disease risk factors, increased levels of SCFA-producing bacteria, and reduced levels of severely harmful bacterial species, including *Escherichia-Shigella* (40). This leads us to suggest that the above bacteria may affect cardiac function through different pathways.

## 5. Conclusion

We found out that the presence of disturbed intestinal flora outstandingly elevated the levels of PAGln, and the correlation among the florae was materially high. Therefore, the metabolites and cardiac function indicators in patients with heart failure suggested that the normal function of the intestinal flora is impaired with heart failure and that pathophysiological changes in the intestine in turn aggravate the development of heart failure. Our study showed a positive correlation between BNP and Pagln. And also found that the more severe the heart failure, the higher the level of Pagln. Consequently, it can be used as an indicator for early screening, diagnosis, and treatment for CHF. In addition, colony transplantation therapy has been used in patients with irritable bowel syndrome, obesity, diabetes, and Alzheimer’s disease, and it may become a new direction for heart failure treatment in the future as this treatment technique matures.

## Data availability statement

The datasets presented in this study can be found in online repositories. The names of the repository/repositories and accession number(s) can be found at: NCBI repository, accession number PRJNA909972.

## Ethics statement

The studies involving human participants were reviewed and approved by Ethics Committee of Shanghai Fengxian District Central Hospital. The patients/participants provided their written informed consent to participate in this study.

## Author contributions

BC and ZZ conducted the investigation and wrote the original draft. BC, ZZ, YS, and HD reviewed and edited the manuscript. HW and ZQ provided the conceptualization and funding. ZQ was the supervisor and conducted project administration. All authors contributed to the article and approved the submitted version.

## Footnotes

1
cloud.magigene.com
